# Accessing microfluidics through feature-based design software for 3D printing

**DOI:** 10.1371/journal.pone.0192752

**Published:** 2018-03-29

**Authors:** Peter G. Shankles, Larry J. Millet, Jayde A. Aufrecht, Scott T. Retterer

**Affiliations:** 1 The Bredesen Center for Interdisciplinary Research, The University of Tennessee, Knoxville, TN, United States of America; 2 The Center for Nanophase Materials Sciences Division, Oak Ridge National Laboratory, Oak Ridge, TN, United States of America; 3 Biosciences Division, Oak Ridge National Laboratory, Oak Ridge, TN, United States of America; University of Illinois at Chicago, UNITED STATES

## Abstract

Additive manufacturing has been a cornerstone of the product development pipeline for decades, playing an essential role in the creation of both functional and cosmetic prototypes. In recent years, the prospects for distributed and open source manufacturing have grown tremendously. This growth has been enabled by an expanding library of printable materials, low-cost printers, and communities dedicated to platform development. The microfluidics community has embraced this opportunity to integrate 3D printing into the suite of manufacturing strategies used to create novel fluidic architectures. The rapid turnaround time and low cost to implement these strategies in the lab makes 3D printing an attractive alternative to conventional micro- and nanofabrication techniques. In this work, the production of multiple microfluidic architectures using a hybrid 3D printing-soft lithography approach is demonstrated and shown to enable rapid device fabrication with channel dimensions that take advantage of laminar flow characteristics. The fabrication process outlined here is underpinned by the implementation of custom design software with an integrated slicer program that replaces less intuitive computer aided design and slicer software tools. Devices are designed in the program by assembling parameterized microfluidic building blocks. The fabrication process and flow control within 3D printed devices were demonstrated with a gradient generator and two droplet generator designs. Precise control over the printing process allowed 3D microfluidics to be printed in a single step by extruding bridge structures to ‘jump-over’ channels in the same plane. This strategy was shown to integrate with conventional nanofabrication strategies to simplify the operation of a platform that incorporates both nanoscale features and 3D printed microfluidics.

## Introduction

Additive manufacturing is poised to change how we design, manufacture, and receive goods [1]. Traditionally, it has allowed engineers and product designers to rapidly produce physical 3D objects in an iterative process to refine ergonomics, identify manufacturing challenges, and communicate marketing concepts rapidly and with minimal cost. The recent availability of a broader range of printable materials coupled with the increased accessibility of lower cost, higher quality printers, and the growth of online innovation and design communities are reshaping how we think about manufacturing and product distribution. Complex, low quantity production parts fabricated by 3D printing have been demonstrated in the aerospace industry [2]. Open source designs for products like prosthetic limbs are being modified and used across the globe [3]. Retailers are even exploring the use of 3D printers for on-demand product customization [4,5].

In the same manner that it has impacted other industries, 3D printing has begun to capture the attention and imagination of the microfluidics community. Additive manufacturing provides an alternative to conventional microfabrication techniques and allows designers to think about fluidic systems in three-dimensions, (e.g. printing unique modular components that can be pieced together to achieve new functions) [6,7]. Issues that had previously slowed the utilization of 3D printing in fluidics such as poor resolution and printer availability are diminishing as printing platforms improve. A recent STL technology has shown internal fluidic channels with dimension as small as 20μm x 18μm [8]. This is minimizing the barriers-to-entry and reducing maintenance costs, thus making 3D printing an attractive alternative to maintaining a conventional cleanroom facility [9].

Direct writing of microfluidic systems with additive manufacturing involves printing the fluidic networks in a resin or thermoplastic so that the channels are fully or mostly enclosed [10,11]. Inlet and outlet ports can be designed so that fluidic connections can be made easily with commercially available parts such as Luer locks or compression fittings [12,13]. Direct-print polypropylene (PP) devices have been successfully used to create custom multi-chamber platforms for organic chemistry experimentation [14,15]. Bhargava et al. demonstrated a parts based system where fluidic components could be printed and assembled much like Lego® bricks to create a fluid network [6]. Further work has been done to develop whole printed devices that integrate off-the-shelf control features such as valves and pumps [16]. However, a major disadvantage of the direct-write method is the surface roughness of the final product. While surface roughness may not significantly impact the flow profile of the microfluidics, it can turn transparent materials translucent, preventing high-resolution imaging [17]. Dolomite Microfluidics has developed a direct write microfluidics platform that is able to create internal channels, but the exterior surface of the device is still cloudy without substantial post processing or the use of embedded glass windows [18,19]. Additionally, many of the resins used are proprietary and biocompatibility and solvent compatibility need to be established for each material [20].

3D printers have also been used to fabricate molds for soft lithography in lieu of SU-8 patterning or silicon deep reactive-ion etching (DRIE) [21]. Masters are created with a 3D printer and the final device material is cast over the design, cured, and removed from the master mold. These molds are primarily used with Poly-dimethylsiloxane (PDMS) [22,23], but have been used with other cast materials such as epoxy [24] or Norland Optical Adhesive (NOA)(Norland Products) [25]. We have previously shown that crude acrylonitrile butadiene styrene (ABS) filaments can be hand-shaped and incorporated into a microfabricated silicon mold to connect individual modules and change the fluidic network for a given application to create fluidic bridges [26], however there is a need for an automated fabrication process for incorporating fluidic bridges into microfluidic systems. Currently, 3D printed microfluidics take less time and require less infrastructure than those created using conventional photolithography, but further advancements are required to make 3D printing a more accessible alternative. Simplifying the design process with more intuitive, application-specific software and implementing a more robust workflow can accomplish this. Groups that want low cost fabrication techniques for quicker turnaround time and teaching purposes can use these techniques to replicate larger microfluidic designs. The FDM process is limited in resolution to a few hundred microns. The highest resolution STL processes can be on the order of 20μm. This remains orders of magnitude larger than photolithography or electron beam lithography techniques. As 3D printing technologies increase in resolution, precision, and extrusion uniformity, the principles in this work will improve on-the-fly microfluidics prototyping and expand the possibilities for creating and using 3D fluidic systems across a broader community.

This work aims to improve the throughput, design process, and optical transparency of 3D printing techniques and strategies for microfluidics. Our feature-based software simplifies the design process by providing a graphical user interface (GUI) for piecing together common microfluidic features into a single custom device. The software’s direct control of the printing order allows for quick iteration of small microfluidic features during the print process and control over the conversion of channels into printer operations in a specific order. This ordering allows optimization of both the resolution and stability of the printed master. A filament deposition modeling (FDM) 3D printer was used to print a master molds. After printing, a solvent annealing process was used to smooth the channels. Subsequently, PDMS was deposited and cured directly on the print bed. The devices utilized the same bonding techniques and connections as conventional soft lithography. A linear gradient generator and two types of droplet generators were fabricated to demonstrate flow stability and the impact of process optimization on the function of these highly utilized designs. Beyond replicating traditional commonly used 2D fluidic designs, 3D printing and layered microfluidics have been used to create bridging structures, but they require either multiple layers of PDMS bonded together [27] or 3D printed support material that has to be removed prior to molding PDMS [24]. Our current process prints three-dimensional bridge structures in a single step that can be used to simplify fluidic networks or reconfigure existing microfabricated designs. This workflow provides a method for rapidly prototyping and replicating microfluidics through a streamlined 3D writing and encapsulation process that is beyond current manual placement techniques.

## Materials and methods

### Feature-based design software

The software GUI and code were developed in Matlab R2015a for Mac (MathWorks) on a MacBook Air computer (Apple, 13-inch, mid 2012, 2 GHz Intel i7, 8 GB 1600 MHz DDR3). The Graphical User Interface Design Environment (GUIDE) plugin was used to layout the GUI of the program. The GUIDE generated a code structure for each button and menu within the GUI. Utility was added to each of the defined functions with Matlab code. The Application Compiler tool was used to package the GUI for distribution so that it could be run on Windows and Mac systems without a full Matlab license. The CAD software has been made available on Github for both Mac and Windows systems (https://github.com/shankles/FluiCAD). The Supplementary folder contains gcode and fig design files for each of the designs used in this manuscript.

### Printer setup

A consumer grade FDM 3D printer (Solidoodle 3, $799 as of 2013) was used to demonstrate the functionality of the Designer software and the fabrication process. Black ABS plastic in a 1.75mm filament (Solidoodle) was used as the mold material with a 0.35mm diameter nozzle at 200°C. The 200mm x 200mm aluminum print bed covered with polyamide tape (Tapes Master) provided an even surface to cast PDMS (Sylgard 184, Dow Corning) molds. ABS mold material and polyamide covering allowed the PDMS to be cast and cured without adhering to the printer or mold. The Solidoodle system heated print bed was set to 85°C to promote mold adhesion during printing and to cure the cast elastomer. Printing was performed with the extruder at 200°C.

### Fabrication process

**Fig 1:**
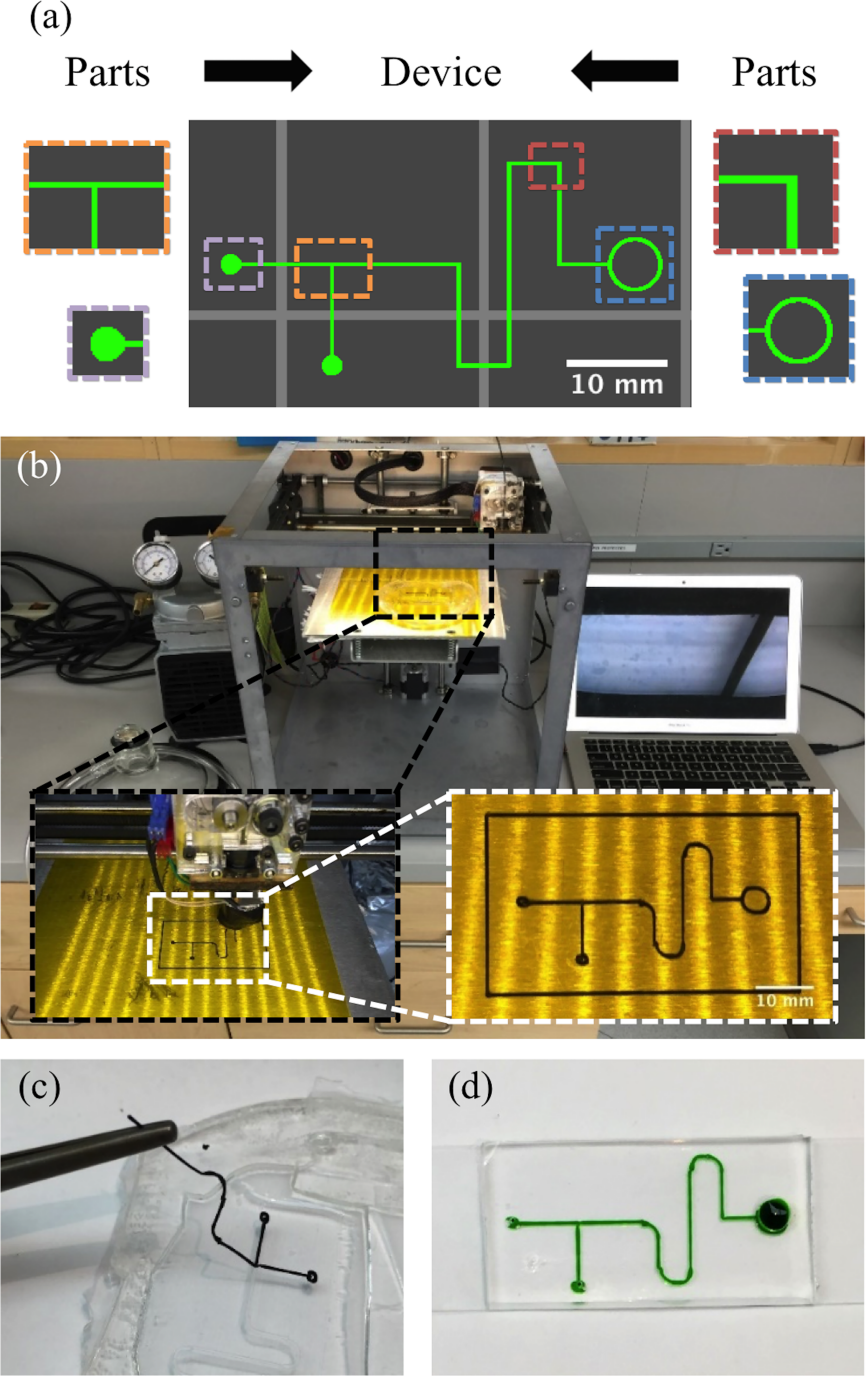
Fabrication process. (a) The device was designed by combining fluidic parts into a custom fluidic network. (b) The design was sent to an FDM 3D printer. (c) The ABS mold is removed from the PDMS device after being cast on the heated print bed. The device was cut into individual devices, and (d) bonded to a substrate for use.

Our feature based design software was used to layout the microfluidic devices. Common parameterized microfluidic features were pieced together to form the final device layout in Fig 1(A). Features were organized into a list showing the print order. The viewing area was used to visualize the layout of the device as it was being assembled. The design was then converted to a g-code file and sent to the 3D printer host software, Repetier Host (Hot-World GmbH & Co.). Repetier can send commands in the g-code to the printer for manually moving the extruder or stage, extruding or retracting the filament, and heating the print bed and hot end. With modest adjustments to the printer parameters in the design software, the host software and g-code are compatible with any 3D printer running Marlin firmware. The host software allowed for final visualization of the g-code prior to printing.

The design was transferred to the printer from the host software over a USB cord and printed in ABS in Fig 1(B). The heated extruder was turned off and the print bed was set to 50°C. At just under the boiling point, acetone was applied to the device molds using a fine-point paintbrush and allowed to evaporate to solvent anneal the surface of the printed channels. The print bed was then turned off and allowed to cool. A PDMS retaining barrier was placed on the print bed around the ABS mold and liquid PDMS resin (10:1 polymer to cross linker ratio) was cast over the mold. A desiccator lid hooked to a vacuum pump (Gast) was placed on the print bed and used to degas the PDMS. After removing all air bubbles from the elastomer, the PDMS was cured directly on the print bed at 85°C for a minimum of 1.5 hrs.

The cured PDMS was removed from the print bed, the devices were cut out with an X-acto knife (Elmer’s), and the ABS mold was removed with forceps in Fig 1(C). At this point the process can be repeated to print additional devices, or an array of devices can be printed simultaneously for higher throughput applications. The remainder of the process follows the workflow of a conventional soft lithography device assembly [28]. Inlets and outlets were punched with dermal biopsy punches (Miltex). The resulting PDMS devices were plasma treated, and can be bonded to glass, PDMS, or silicon substrates. Fig 1(D) shows the device bound to a glass slide and filled with food coloring to show the channels.

### Droplet generator

Two types of droplet generators were constructed as a proof-of-concept for the printing method. A T-junction device and a flow focusing design were chosen based on previous micro-scale work [29–33]. Fluorescein dye (10mM in PBS, Life Technologies) was the aqueous phase and mineral oil was the oil phase. The fluorescein dye was injected at a constant rate (1μl/min), while the mineral oil was modulated (2μl/min to 20μl/min) to control droplet size. Epifluorescent images were taken using a Nikon Ti-U inverted microscope with a FITC filter. The images were analyzed in ImageJ to quantify droplet length within the channel [34].

### Gradient generator

A three-step linear gradient generator was fabricated to test control of fluidic connections. From two inlets, microchannels divide three times to produce five channels that recombine and diffuse in a single channel to form a gradient of the two inlet solutions [35,36]. The concentrations recombine in a main channel and diffusion evens out the concentrations, forming a continuous gradient [37]. Fluorescein dye (10mM in PBS) and PBS were used to characterize the operation of the device. The inlets had a balanced flow rate of 0.5μl/min in each. After equilibrium was reached, epifluorescent images were taken on a Nikon Ti-U inverted microscope with a FITC filter cube at each of the channels prior to mixing within the large channel. Epifluorescence images were analyzed in ImageJ to measure the maximum intensity of the fluorescent dye within each channel.

### 3D microfluidics

By controlling the extrusion paths of the 3D print head, 3D microfluidics can be fabricated using a bridge structure in a continuous, vertical extrusion process rather than conventional layer-by-layer deposition. The initial portion of the 3mm long bridge feature (600μm diameter) is an extruded filament created in the vertical direction away from the print bed, this is allowed to solidify for 3sec prior to completing the bridge by drawing the filament from the top of the vertical post to a user-specified final position on the print bed. The resulting bridging structure is a right triangle with filament spanning the points specified. A “braid” of three channels was fabricated with overlapping channels to demonstrate functionality. The braid printing is demonstrated in S1 Video. The bridge structure is limited to a single filament width (400μm diameter). After casting and curing PDMS over the device, the bridge structures can be removed by pulling on the exposed filament. The weakest point at the top of the bridge separates and the ABS mold can be removed in two pieces from either opening leaving open channels through the PDMS.

The bridge structure was combined with microfabricated masters to create multiscale devices. The silicon master contained nanoscale features similar to ones demonstrated previously [38]. A thin layer of PDMS was spin-coated onto the master (500rpm for 45sec), vacuum degassed, and cured at 75°C for 30min. A fluidic architecture was created in the design software to simplify the network of the silicon master, bridging together inlets and outlets. 3D printed channels were fabricated and cast to form a PDMS replica. The 3D printed PDMS layer was then bonded to the coated wafer to form a multilayer device. The PDMS-to-PDMS plasma-bonded device was baked at 75°C for 10min then removed from the wafer. The inlets and outlets connected by 3D structures were opened with an X-acto knife and the other inlets and outlets were punched with a dermal punch. The PDMS was plasma-bonded to a glass slide to complete the assembly. With this method, the fluid network of the device can be altered without additional nanofabrication steps.

## Results and discussion

We created an integrated workflow built on a feature driven design platform to produce functional fluidic platforms for common microfluidic applications including gradient generation for chemotaxis and other cellular studies, droplet generation for single cell analysis and small volume reactions, as well as 3D microfluidics that can be used to form complex fluidic architectures and simplify fluidic networks for microfabricated designs by allowing for overlapping channels in a single step. To overcome limitations (lower resolution, surface roughness, and low transparency) [39,40] of FDM-produced microfluidics, we employ 3D printing with an acetone finishing step to create a smooth microfluidic master for fabricating transparent, 3D microfluidics. By solubilizing the surface of the ABS print with acetone, the surface is chemically polished without greatly altering the channel geometry. The smooth ABS prints were used as molds to cast PDMS devices. Using the heated print bed on the printer, the casting process was done directly where the device was printed, reducing the likelihood of deformation and breakage.

### Design software operation

The feature-based design software was developed in the Matlab GUIDE environment, and the final program packaging was achieved with the Application Compiler, allowing it to be run on other computers without a full Matlab license. Common 3D printed microfluidic techniques often use CAD software to design fluidic networks. CAD programs provide powerful tools for design, but training and maintenance costs for professional packages are limiting factors. Completed CAD design files are imported to a slicer program that processes the geometry into g-code to be used with the 3D printer. With our feature based design the process is simplified by giving the user a list of parameterized microfluidic features to choose from in order to build a custom fluidic system. The program writes the g-code to print the device directly. This allows the user to correct any writing problems quickly by changing the printing order, unlike typical slicer software.

**Fig 2:**
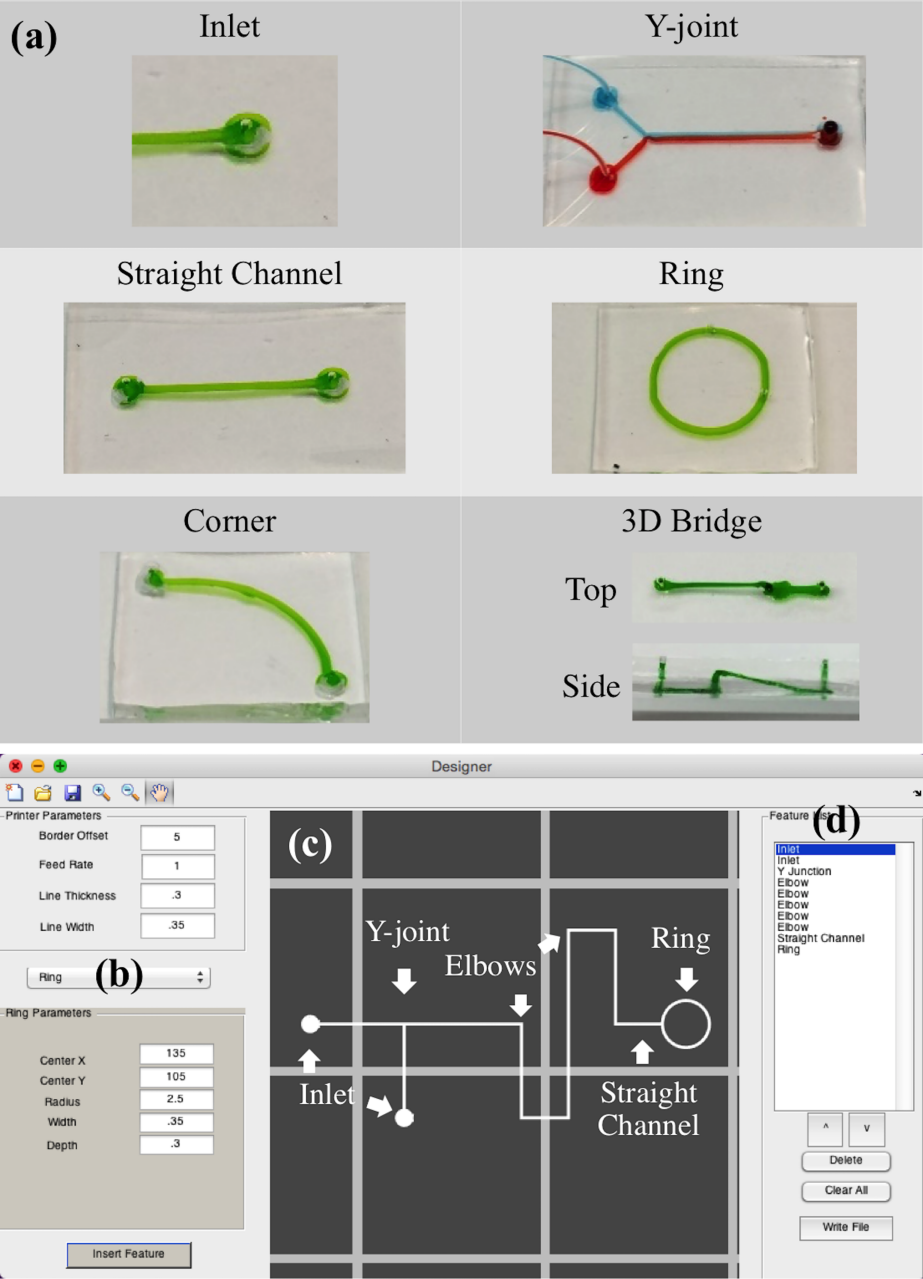
Feature parameters and program GUI. (a) Table of features available for the design process. The GUI consists of 3 sections (b) the printer and feature parameters are given, (c) the design is represented graphically, and (d) the parameter list of all the parts in the current design for editing.

The GUI of the program is organized into several operational blocks. The printer parameters are set based on the printer selected for use and the print resolution. The required feature is selected from a dropdown window and the user defines the associated parameters Fig 2(B). The feature is added to the visual area Fig 2(C). The visual area is a graphical representation of the entire print bed. The inserted feature is also added to the feature list at the right side of the GUI Fig 2(D). Features making up the current device can be reordered or replaced with different parameters to improve the printing process. From the feature list, the print order can be changed to quickly correct printing problems. Individual features can be deleted, and the entire feature list can be cleared.

The list of features includes an inlet for tubing connections, a straight channel, an elbow for sweeping corners, a y-intersection to join and separate channels, a ring feature that can be used for radial patterns, and a bridge structure to overlap channels. The parameters to construct each of these parts are based on the coordinate system of the 3D printer bed. Parameters such as starting position and channel dimensions are used to construct each part. S1 Fig shows the list of features available and all the parameters required for each.

With the designs completed, the “write” button creates g-code for each part in the feature list to replicate the device on a 3D printer. The printer parameters and a barrier around the device are written first. The barrier acts to prime the extruder and reveals errors in the print bed calibration (poor adhesion from the print head being too far away or flat or split channels from being too close to the print bed). The features are then written to the file. The Matlab code works through the feature list, writing g-code based on the type of channel being printed and the parameters of the printer and the individual devices. This g-code can be visualized and sent to the printer using a 3D printer host program. The designs of each of the devices featured in this work are provided in S2 Fig.

### Printing process

**Fig 3:**
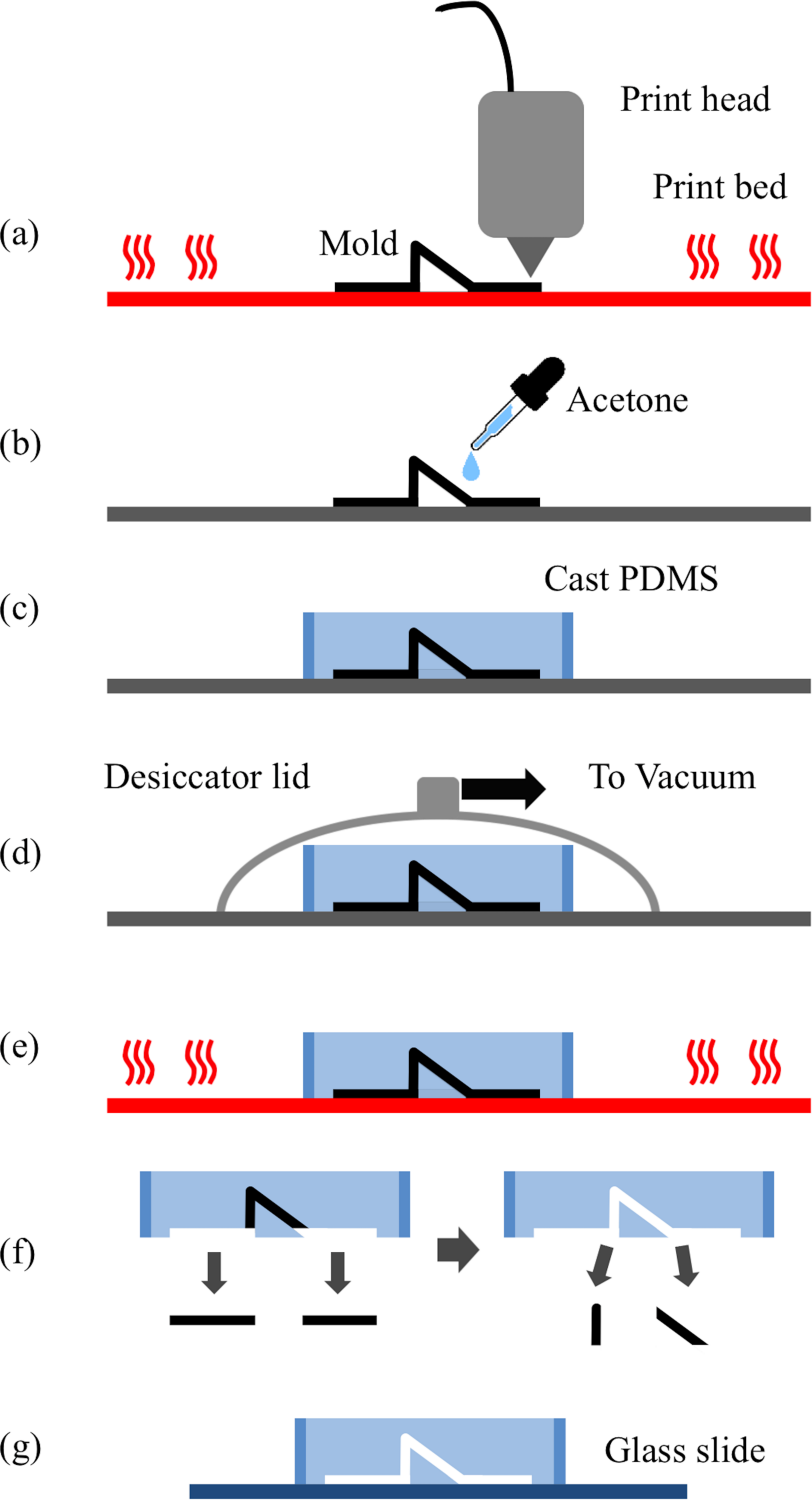
Fabrication process diagram. (a) The device was printed on a heated print bed. (b) Acetone was applied to the surface of the device to anneal the ABS surface. (c) PDMS was cast over the mold, (d) a vacuum degassed the PDMS, and (e) the heated print bed cured the device. (f) The device was removed from the bed and ABS mold and (g) bound to a glass slide.

The 3D printing process focuses on efficient use of the printer’s capabilities to print a mold to form channels in PDMS elastomer. The resulting device is a PDMS device that functions in the same manner as those made in traditional soft lithography using silicon masters. The 3D printer used dictates the feature resolution. The Solidoodle printer was able to fabricate channels with consistent results between prints. The characterization results for the Solidoodle printer used are shown in S3 Fig. By directly writing the microfluidic channel master, designs can be replicated in PDMS, and bonded to glass within 3hr. Fig 3 summarizes the fabrication process. By comparison, the conventional process of fabricating a mold with photolithography using SU-8 or DRIE dry plasma etching of silicon wafers can take several days or weeks if new photomasks have to be ordered rather than fabricated on site.

The resulting printed designs were tested for accuracy by printing a series of straight channels with increasing numbers of filaments in width and height. For a single filament extrusion, the channels were on average 180μm deep and 940μm wide with a standard deviation of 2μm and 14μm respectively. As the number of filaments increase in the width of a channel the width increased by 470μm (n = 3, SD ± 240μm). Stacking layers to increase the channel height adds an average of 270μm (n = 3, SD ± 46um) in channel height. Using a smaller diameter extruder tip can potentially reduce these incremental dimensions. From the user defined channel height and width, the feature based design software divides the channel into the correct number of filaments to have a channel width and height as close to the designed value as possible.

**Fig 4:**
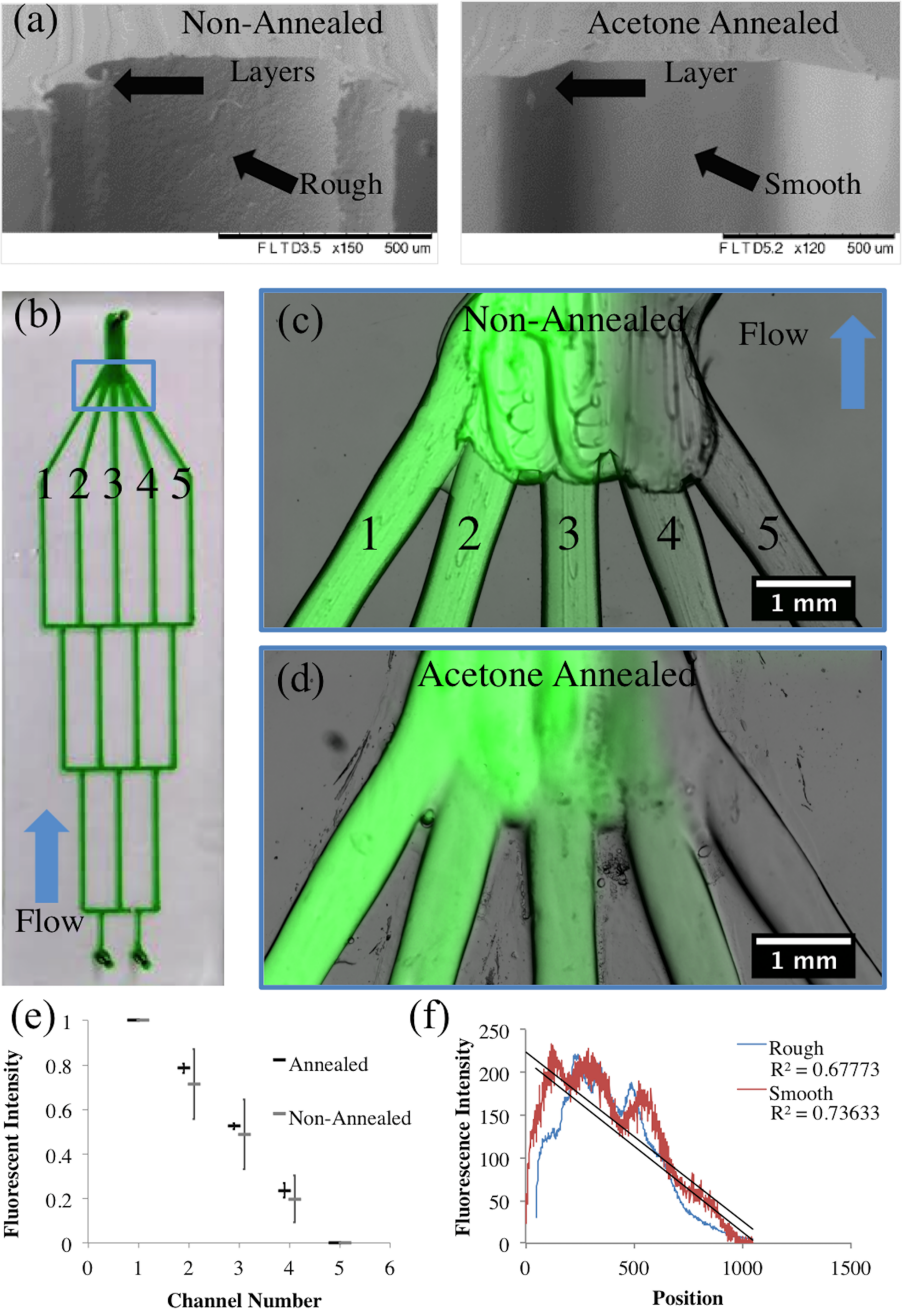
Acetone annealing gradient generators. (a) SEM images show the surface of the ABS mold annealed by applying acetone. (b) A microfluidic gradient mixer produced using our ABS mold printing process. (c-d) Images of the device show dilution channels recombining. (c) Annealing smooths the surface for more even imaging. (d) Non-annealed device shows rough surfaces from the printing process. (e) The maximum fluorescent intensity from the individual channels prior to rejoining shows greater variability in non-annealed devices. (f) The fluorescent intensity profile in the channels after recombining show the gradient forming. Variation in the chamber height from 3D printing causes variation across the profile deviating from the expected linear gradient.

Layers of filament that make up the channels were clearly visible and had a rough surface after printing. Surface roughness of the channels was minimized through an acetone solvent annealing treatment. The annealing process was adapted from common 3D printing techniques that use solvents to solubilize the surface of a printed model to smooth out the layering effect of FDM 3D printing. Printed models are exposed to an acetone vapor, within a closed container, to dissolve and smooth the surface. For our process, a small amount of acetone was applied to the surface of the printed channels using a fine-tip paint brush and allowed to evaporate with the print bed set to 50°C to accelerate the process. Temperatures > 50°C caused bubbles in the ABS as the acetone evaporates. Ultimately, this process removes roughness in the surface and smooths individual layers from the printing process Fig 4(A) this improves the optical properties and makes the flow resistance of the channels more uniform.

### Applications

To demonstrate the utility of direct write microfluidic designs and 3D microfluidics using our feature-based design utility, common fluidic architectures were designed, printed, assembled, and tested. A gradient generator was fabricated to test the replication of the printing process. Uneven resistance within the bifurcating channels of a gradient generator will cause variations in the concentration gradient, indicating variations between the identically designed channels. The device worked by splitting and recombining channels to form combinations of the two inlet solutions. The five channels recombine in a larger channel where diffusive mixing makes a continuous gradient. Solvent annealing the channels removed microscale irregularities that interfere with laminar flow and uniform gradient formation (Fig 4A). Fluorescence intensity profiles (Fig 4E) show a linear decrease in intensity across the combined flow as a result of combinatorial mixing. Line profiles of the fluorescence in each channel were taken where the numbers indicate in Fig 4B prior to recombination in the final channel. The concentrations of fluorescein in the annealed device show lower variability between devices demonstrated by the standard deviation. The annealed channels had a maximum standard deviation of 0.032, N = 3, and the non-annealed channels had a maximum standard device of 0.16, N = 3. The fluorescence gradient after recombination in the large channel is shown in Fig 4F. The device that was acetone annealed is a better fit to a linear gradient, but the varying thickness of the chamber complicates the optical measurement unlike single filament channels prior to joining together.

**Fig 5:**
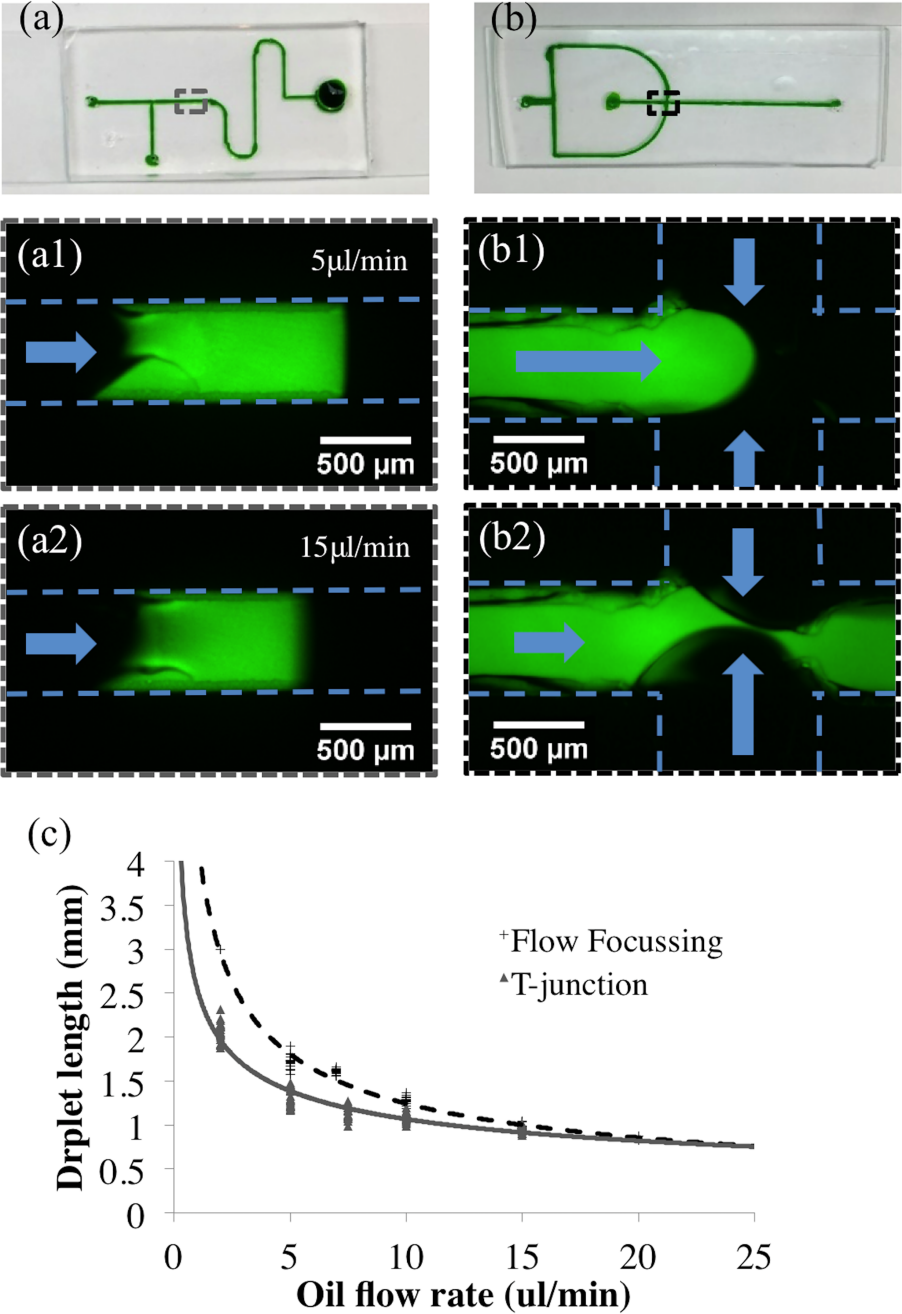
Droplet generators. (a) The T-junction device was operated with the fluorescein flow rate at 1μl/min and the oil at (a1) 5μl/min and (a2) 20μl/min. (b) The flow-focusing device operates with the same flow rates. (b1-2) show the oil channels pinching off a droplet from the fluorescein channel. (c) Formed droplets are highly replicable and can be controlled by altering the oil flow rate from 2μl/min to 20μl/min.

Two designs for a droplet generator using both a flow focusing design and a T-junction device were fabricated. Even with larger channel sizes than typical microfluidic droplet generators, the 3D printed designs were able to form droplets consistently. Fig 5A and 5B shows the completed chips bonded to glass and filled with food coloring as well as the flow-focusing device forming droplets as the oil phase shears off droplets of fluorescein dye. The droplets formed in both devices where shown to vary in size as the flow rate of the oil phase was modulated from 2μl/min to 20μl/min with a constant flow rate of the aqueous phase at 1μl/min. Droplets formed at 20μm/min had a volume of 0.14μl. These are similar to other droplets formed using 3D printing techniques[6].

### 3D microfluidics

**Fig 6:**
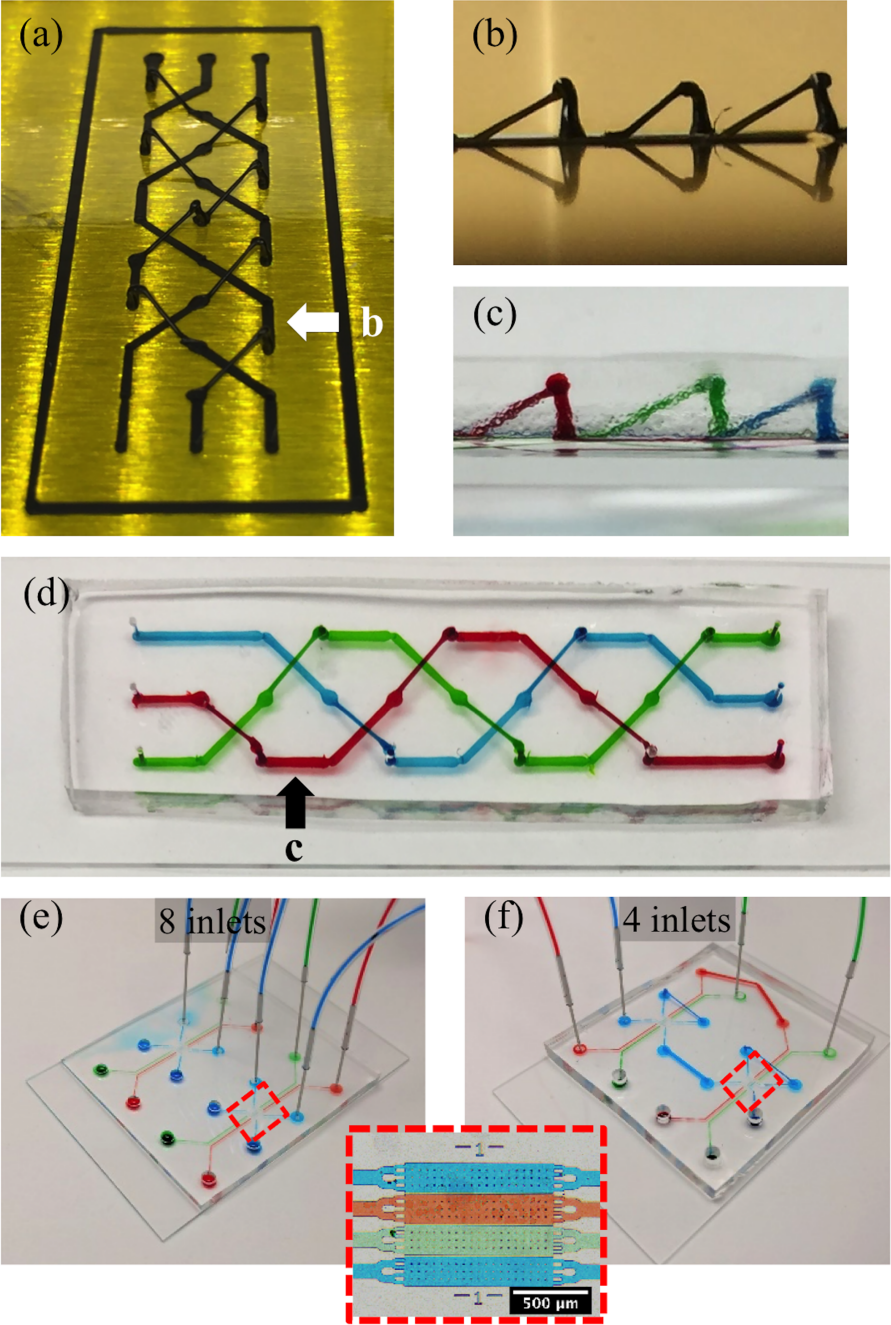
3D microfluidics. Using 3D capabilities of the feature-based software, bridges were printed to create an overlapping design with three channels from an offset (a) and side (b) view. (d)Top view—overlapping channels remain separate from one another. (c) Side view—the bridging structure raises off the plane of the glass slide. The expanded view shows the printing direction for the bridging structures. (e) The microfabricated structure along with an inset of the chambers with each channel independent of one another. (f) Shows 3D printed structures connecting channels and overlapping to simplify the device control.

The power of using 3D printers is the ability to create microfluidics that are free to move in all spatial dimensions rather than the planer construction of conventional techniques. Design aspects of 3D printing allow for the fabrication of common designs as well as designs that have unique architectures, however, traditional slicer programs limit the design capabilities by building a device out of multiple layers of material. The feature-based design software writes paths in the z-direction continuously to form structures. This ability was used to create bridges that effectively suspend channels over the printing surface. Fig 6 shows bridges created in a braid pattern so that channels can pass across one another without being connected. Extruding a pillar to a height of 3mm, allowing the ABS to solidify for a short time, and extending a filament diagonally down to the final position forms 3D bridge structure. The ABS printed mold is shown in Fig 6(A) with a side view 6(B). After casting PDMS over the structures, the ABS molds were removed with tweezers leaving internal channels without multiple layers of PDMS. The resulting device was bound to a glass slide to complete the fabrication Fig 6C and 6D.

In order to take advantage of the 3D printing without the drawback of lower resolution, 3D printed fluidic networks were combined with microfabricated architectures. These multiscale devices have micro and nanoscale chambers that are routed to one another with 3D printed channels. By 3D printing new networks, the microfabricated devices can be repurposed for multiple experiments without further cleanroom fabrication. Bridges can be incorporated into the design to overlap network channels and simplify fluidic control of the device. Fig 6(E) shows a microfabricated device with eight inlets and eight outlets to individually control each of the four channels of the two devices. Fig 6(F) shows the completed bridged device with the blue and red channels being controlled through a single inlet and outlet. The two green channels remain independent and can be changed between the devices, holding the other chambers constant. This structure can be adjusted to allow for changes in the operation of the device without further cleanroom fabrication.

## Conclusions

The feature based design software and associated method for direct molding PDMS microfluidic devices using an FDM 3D printer was shown to be able to fabricate frequently used microfluidic devices, as well as complex 3D designs that photolithography or micromachining are not capable of. By increasing the accessibility of 3D printed microfluidics, the number of applications and user base of microfluidics can be broadened. This technology could be adapted to academia for teaching the basics of microfluidics by taking advantage of the ease of use and low cost of the approach. Additional work to develop teaching modules that adjust to the time required to iterate through multiple designs. Our feature-based design utility allows researchers to fabricate microfluidics quickly without the need for cleanroom facilities.

The software interface described here was developed to simplify the design process by giving the user a list of common microfluidic building blocks that can be combined into novel fluidic architectures. The software controls the conversion of the design to g-code to improve control of the printing process. Printing was done using a Solidoodle 4 3D printer, but the Matlab code can be used with any FDM 3D printer running the Marlin firmware. This technique fabricates devices in less time, with lower costs, and with similar results to conventional soft lithographic techniques. The process was shown to be a good alternative to soft lithography and can be integrated with micro and nanofabricated devices to reconfigure systems through 3D fluid networks.

## Supporting information

S1 Video 3D bridge printing process. The printer extrudes posts, allows them to solidify, and suspends a filament from the post to the print bed. The process is done continuously rather than layer by layer.
(MP4)

S1 Fig Feature parameters and part list. Printer parameters for each parameter are shown in (a). Adding parts to a design populates the graphical area (b) as well as the Feature list (c). The order of parts in the list indicates the printing order.
(TIF)

S2 Fig Device designs used. The completed designs for each device used are shown in the Matlab design environment. The droplet generator (a) and gradient generator (b) show replication of common microfluidic designs. The second droplet generator design is not shown. The coil design (c) and the network architecture (d) were used to show the 3D capabilities of the printing process.
(TIF)

S3 Fig Printer characterization from the Solidoodle printer used. The number of layers do not affect the width of the channel (a), but the number of lines can affect the height of the channel. The smallest channels were roughly 1mm wide and 200μm in height.
(TIF)
