# Supplementary figures and images for: Accessing microfluidics through feature-based design software for 3D printing
Source: PLoS One. 2018 Mar 29;13(3):e0192752. doi: 10.1371/journal.pone.0192752 (PMC5875762; doi:10.1371/journal.pone.0192752)

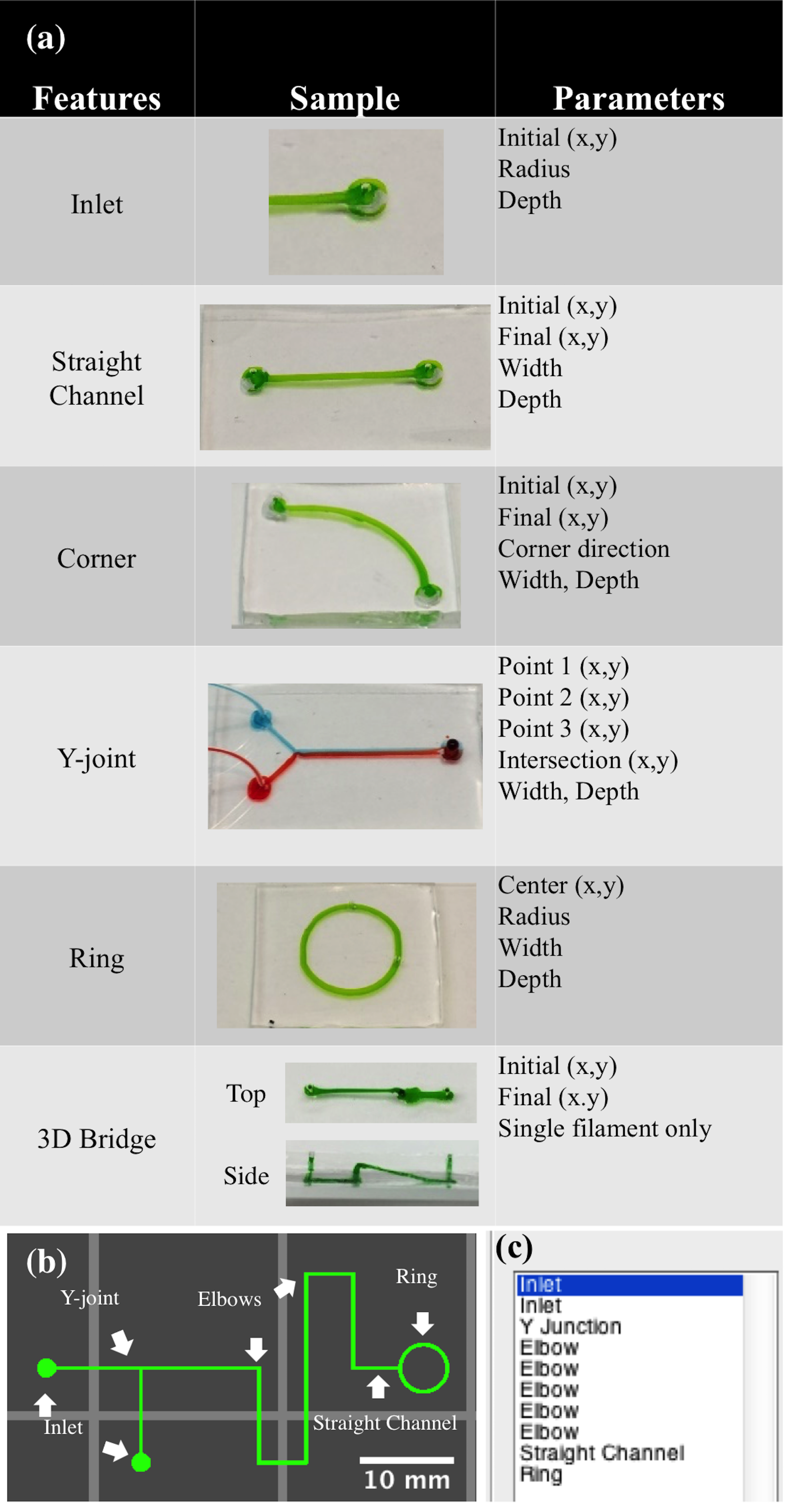

Supplement: S1 Fig — Printer parameters for each parameter are shown in (a). Adding parts to a design populates the graphical area (b) as well as the Feature list (c). The order of parts in the list indicates the printing order. (TIF) [file pone.0192752.s002.tif]

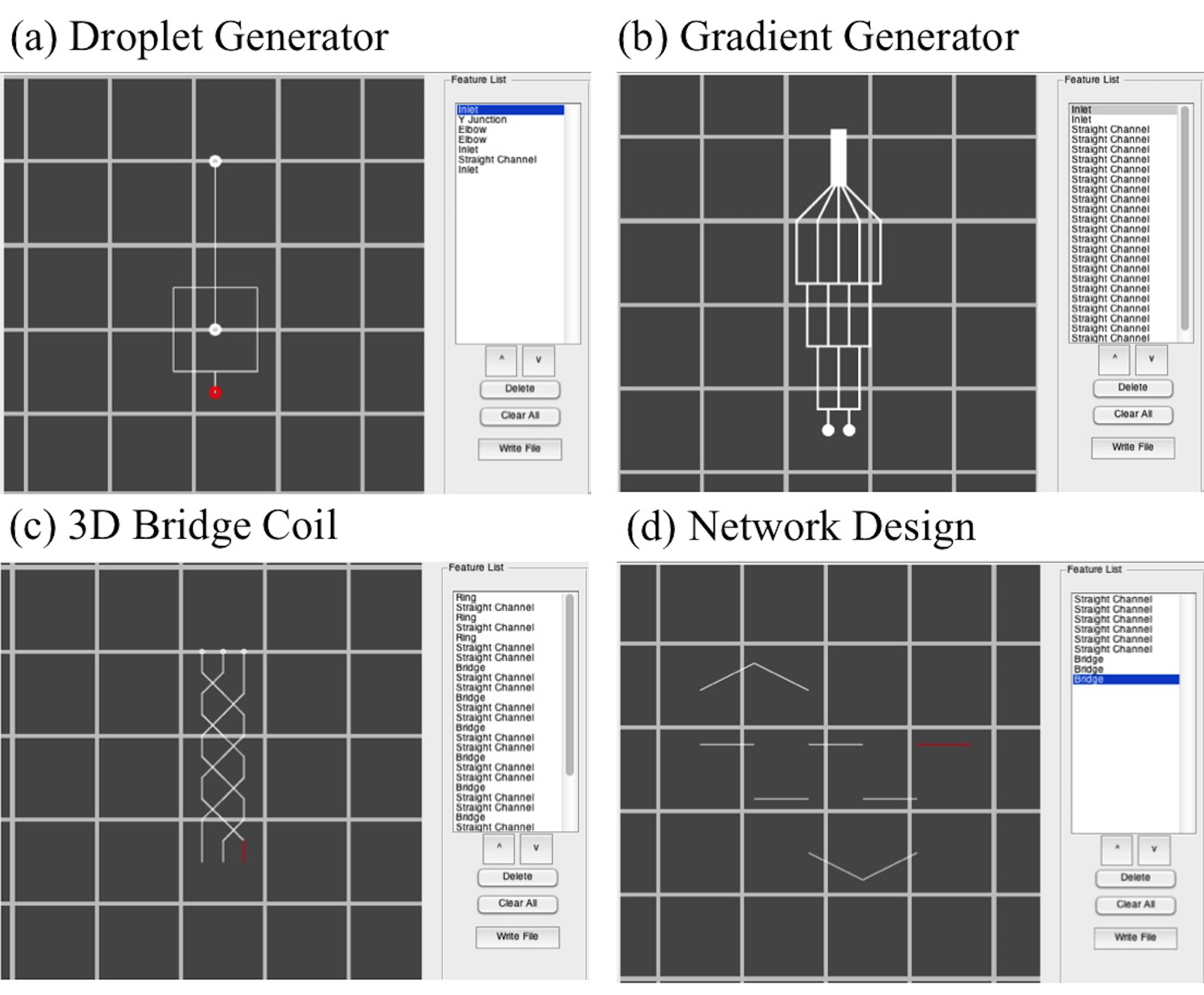

Supplement: S2 Fig — The completed designs for each device used are shown in the Matlab design environment. The droplet generator (a) and gradient generator (b) show replication of common microfluidic designs. The second droplet generator design is not shown. The coil design (c) and the network architecture (d) were used to show the 3D capabilities of the printing process. (TIF) [file pone.0192752.s003.tif]

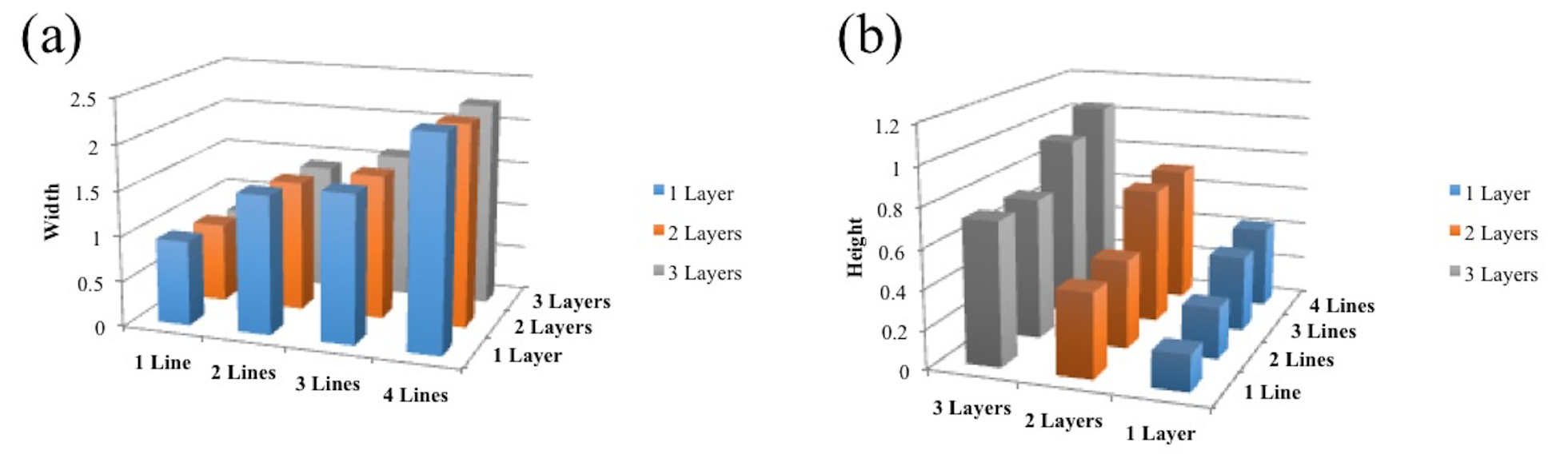

Supplement: S3 Fig — The number of layers do not affect the width of the channel (a), but the number of lines can affect the height of the channel. The smallest channels were roughly 1mm wide and 200μm in height. (TIF) [file pone.0192752.s004.tif]
